# Risk of new onset and persistent psychopathology in children with long-term physical health conditions: a population-based cohort study

**DOI:** 10.1007/s00787-023-02170-3

**Published:** 2023-02-28

**Authors:** Laura Panagi, Simon R. White, Xiaolu Dai, Sophie Bennett, Roz Shafran, Tamsin Ford

**Affiliations:** 1 Department of Psychiatry, University of Cambridge, The Clifford Allbutt Building, Biomedical Cambridge Biomedical Campus, Hills Road, Cambridge, CB2 OAH UK; 2 UCL Great Ormond Street Institute of Child Health, 30 Guilford Street, London, WC1N 1EH UK; 3 Department of Social Work and Social Administration, The University of Hong Kong, Jockey Club Tower, Centennial Campus, Pokfulam, Hong Kong

**Keywords:** Children, Long-term physical health conditions, Predictors, Mental health conditions, Follow-up

## Abstract

Children and young people (CYP) with long-term physical health conditions (pLTCs) have increased risk of psychopathology compared to physically healthier peers. We explored risk factors for new onset and persistent psychiatric disorders in CYP with pLTCs compared to CYP without pLTCs. This 3-year follow-up study involved a UK representative sample of CYP from the British Child and Adolescent Mental Health Surveys (*N* = 7804). We examined potential baseline predictors of new onset and persistent psychiatric disorders at follow-up in four groups of children based on the presence of any physical and/or any psychiatric conditions at baseline. Psychiatric disorders were assessed using standardised multi-informant diagnostic assessment. Separate multivariable binary logistic regressions were conducted for each group. In CYP with pLTCs, rented housing (aOR = 1.42, 95% CI  1.01 to 1.99), non-traditional family structure (aOR = 2.08, 95% CI  1.42 to 3.05), increased parental distress (aOR = 1.09, 95% CI  1.04 to 1.14), and greater peer relationship difficulties (aOR = 1.29, 95% CI  1.19 to 1.39) predicted future psychiatric disorder. Only peer relationship difficulties predicted persistent disorder (aOR = 1.27, 95% CI  1.17 to 1.38) in this group. A greater number of factors predicted the onset of psychiatric disorder in CYP with pLTCs compared to physically healthier peers and similarly, a higher number of factors predicted persistent disorder in CYP without pLTCs. CYP with pLTCs might comprise a group with different vulnerabilities, some of which are potentially tractable and may be useful indicators of patients who require preventable or management interventions.

## Introduction

An extensive literature demonstrates that children and young people (CYP) with long-term physical health conditions (pLTCs) experience approximately a fourfold greater risk of psychopathology compared to the general population of CYP [1–12]. Our previous research with the 2004 British Child and Adolescent Mental Health Survey (BCAMHS) demonstrated that compared to children without a LTC, CYP with any LTC had higher SDQ total difficulties scores at baseline (adjusted mean difference 1.4, 1.1–1.6) and follow-up (1.1, 0.8–1.4), and were more likely to have a psychiatric disorder at baseline (adjusted odds ratio [aOR] 1.59, 1.34–1.89) and follow-up (1.75, 1.44–2.12) [13]. Considering the high rates of physical-mental health comorbidity in CYP, previous research has highlighted the importance of developing integrated approaches toward mental and physical health [14, 15]. Identifying the risk factors for the onset and persistence of mental health disorders in CYP with pLTCs could help (a) detect potential aetiological processes, (b) recognise high-risk cases to be targeted for early intervention services, and (c) identify factors that interventions should aim to influence. However, most CYP with pLTCs do not display significant psychopathology [2], which raises the question: What are the differences between those who do and do not develop mental health disorders?

Age and sex [3, 4] differences in the risk of psychopathology have been reported in meta-analyses of cross-sectional studies involving CYP with different pLTCs. In a large-scale, cross-sectional study carried out in childhood cancer survivors, Black ethnicity was associated with better mental health outcomes after adjusting for socioeconomic status [5]. Another cross-sectional study in children with cancer reported that those with longer hospital admissions had more post-traumatic symptoms [6], and post-traumatic symptoms were correlated with poorer self-reported family functioning in a recent meta-analysis including CYP with different pLTCs [7]. Moreover, two cross-sectional studies in paediatric cancer patients demonstrated that intellectual disability was positively associated with symptoms of depression [8] and anxiety [9].

Although informative, previous studies in CYP with pLTCs are often limited by their cross-sectional design [5, 6, 8, 9], the inclusion of small, clinical samples [6, 8, 9] that are likely to comprise the most severely affected CYP, and the application of self-reported questionnaire-based data [5, 6, 8, 9] rather than standardised diagnostic assessments of mental health.

A greater body of cross-sectional and longitudinal evidence has assessed risk factors that influence mental health in the general population of CYP. Several child, parent, family, and school factors have been implicated in the onset or the progression of psychopathology including peer relationship difficulties [10, 11], parental socioeconomic status [11, 12], parental mental distress [16], living in a single-parent family [17], poor family functioning [11], school absenteeism [18], poorer educational attainment [19], and intellectual disability [11].

CYP with pLTCs might comprise a distinct group with different vulnerabilities. As mentioned earlier, identifying the risk factors related to the onset and persistence of mental health disorders in CYP with pLTCs is essential to improve detection and timely intervention. However, no previous studies have explored the risk factors for CYP with pLTCs using population-based rather than clinical samples. We aimed to explore candidate risk factors for new onset and persistent psychiatric disorders in CYP with pLTCs compared to those without pLTCs using two population-based longitudinal datasets. Our research questioned whether the predictors of new onset and persistent psychopathology differed between CYP with pLTCs as compared to those without them.

## Methods

### Participants

The British Child and Adolescent Mental Health Surveys (BCAMHS) comprise two comparable population-based surveys of CYP aged 5 to 16 years, conducted in the UK in 1999 (*N* = 10,438) and 2004 (*N* = 7977). The samples were recruited via the Child Benefits Register; detailed survey design information is presented in Online Resource 1. Baseline surveys were repeated in the same samples in 2002 and 2007, respectively. The combined baseline sample and their 3-year follow-ups were analysed in this study. The original BCAMHS were approved by the Medical Research Ethics Committees [20]. Specific ethical approval was not necessary for this study as no additional participant contact was required for this secondary analysis; the data are available via application to the UK Data Service.

**Fig. 1:**
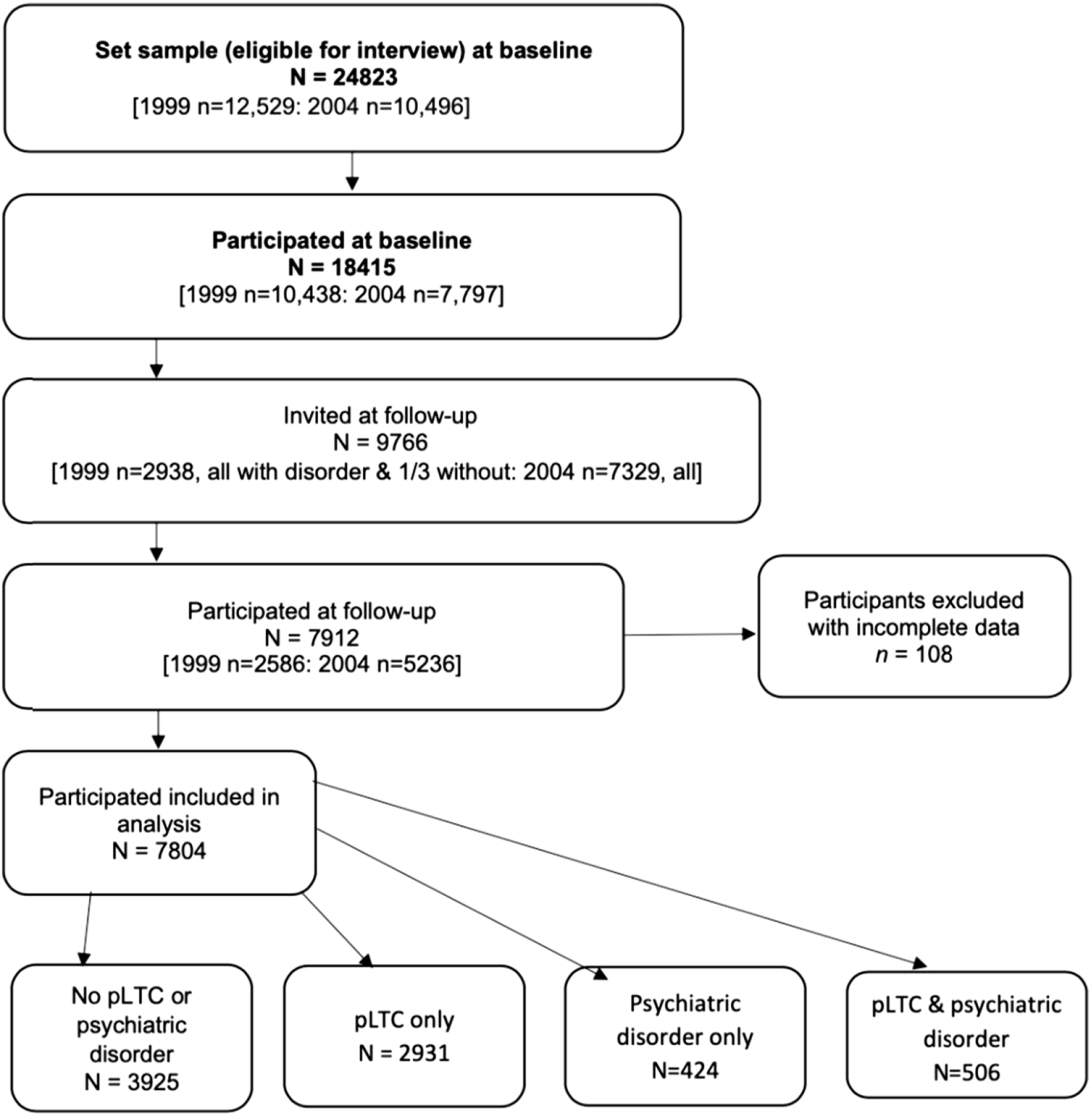
Inclusion of participants from the 1999 and 2004 BCAMHS surveys for analysis

A study flow diagram is depicted in Fig. 1. Information was collected from parents (the natural mother in 94% of cases), young people (aged ≥ 11 years), and teachers (the family nominated a teacher who knew the child best). Parents and young people were interviewed face-to-face, using computer-assisted interviews by trained interviewers. Self-reported questionnaires were also completed by all informants. We excluded participants with incomplete baseline or follow-up data (*n* = 108), resulting in an analytical sample of *N* = 7804.

### Measures

#### Any pLTC

The parent was shown a list of conditions and asked to report whether their child currently had any of them; parents had to the option of reporting and describing additional conditions but no additional details were sought. Parents reported on the following conditions (yes/no): asthma, eczema, hay fever, epilepsy, cerebral palsy, muscle disease, co-ordination problems, heart problems, food allergy, kidney/urinary tract problems, a condition present since birth (e.g. club foot or cleft palate), deformities, spina bifida, cystic fibrosis, blood disorders, missing limb(s), diabetes, cancer, vision problems, or hearing problems. We formulated which conditions to include in discussion with the Study Steering Committee, which comprised academic and practicing paediatricians’ and mental health practitioners. Our discussion was based on the consensus definition of pLTCs as reported by Mokkink and colleagues [21], which resulted in the exclusion of the following conditions either because they were mostly mild and self-limiting (glue ear or otitis media; having grommets;, developmental problems (bed wetting, soiling); too imprecisely described (obesity, stomach or digestive problems or abdominal or tummy pains, non-food allergies) or too closely associated with mental health (migraine or severe headaches, Chronic Fatigue Syndrome or Myalgic Encephalomyelitis [13]). Some conditions met more than reason for exclusions; for example, recurrent abdominal pain may be related to underlying emotional difficulties, but equally to abdominal pathology of highly variable severities. We excluded obesity from both the initial and sensitivity analyses due to very low numbers of children reported as obese (< 50), which probably relates to the observation that parental report greatly underestimates actual levels of obesity [22].

#### Any psychiatric disorder

The Development and Wellbeing Assessment (DAWBA) was used to assess psychiatric disorders according to the Diagnostic and Statistical Manual (DSM IVr) of Mental Disorders [23, 24]. The DAWBA is a standardised diagnostic assessment that combines highly structured questions about various disorders with semi-structured probes, incorporating information from parents, young people, and teachers. Both structured and qualitative information from all informants were reviewed by clinicians who assigned diagnoses based on DSM IVr criteria [23]. The DAWBA covers various types of Anxiety, including Post Traumatic Stress Disorder and Obsessive Compulsive Disorder; Autism Spectrum Conditions; Attention Deficit Hyperactivity Disorder; Affective, Behavioural, Tic and Eating Disorders [20]. When first evaluated, the DAWBA demonstrated excellent discrimination between community (*N* = 491) and clinic (*N* = 39) samples [23]. CYP in the community sample with and without diagnosed disorders differed markedly in external characteristics and prognosis. There was substantial agreement between DAWBA and case note diagnoses in the clinic sample, although the DAWBA diagnosed more comorbid disorders [23]. Chance corrected agreement for two clinical raters independently rating the same 500 CYP from BCAMHS 1999 0.86 for any disorder (SE 0.04), 0.57 for internalizing disorders (SE 0.11), and 0.98 for externalizing disorders (SE 0.02) [25]

#### Predictor variables

Predictor variables were selected based on previous research indicating their effect on mental health in CYP. These were age (years), sex (male/female), ethnicity (White/Black/Indian, Pakistani or Bangladeshi/other), hospitalisation because of serious illness (yes/no), peer relationship difficulties (score; see below), parental employment status (active/inactive), housing tenure (own/rent), family structure (traditional/blended/lone parent), parental mental distress (score; see paragraph below), and family functioning (health/unhealthy; see paragraph below). In the main analysis, all predictor variables were measured at baseline using parental report, as the latter was available for the entire sample. A sensitivity analysis substituted self-report and teacher report for parental peer relationship difficulties. Self-report was only available for CYP aged 11 years or more, while not all families agreed to teacher involvement and not all invited teachers participated.

Peer relationship difficulties were assessed with the 5-item peer subscale of the Strengths and Difficulties Questionnaire, which covers isolation, friendship, popularity, victimization, and the ability to relate to children as compared to adults [26]. Parents and young people aged 11 or over responded to each of the items on a scale from 0 (not true) to 2 (certainly true). Total scores range from 0 to 10, with higher scores indicating increased difficulties. The internal consistency of the peer relationship difficulties subscale was acceptable in this sample (Cronbach’s alpha [*α*] = 0.60).

Parental mental distress was indexed using the 12-item General Health Questionnaire [27], which assesses common psychological problems over the last two weeks. Total scores range from 0 to 12, with higher scores indicating elevated distress. The internal consistency of this measure was excellent in our sample (*α* = 0.88). Family functioning was measured using the 12-item McMaster Family Assessment Device [28], which provides statements about current family behaviour, such as “in times of crisis, we can turn to each other for support” and “individuals are accepted for what they are”. Response categories ranged from 1 (strongly agree) to 4 (strongly disagree). The summed score was divided by 12 with final score ranging from 1 to 4, where higher scores indicated poorer functioning. A binary measure was created (healthy/unhealthy) using a cut-off point of 2 [29]. The internal consistency of this scale was very good in this sample (*α* = 0.84).

School factors were also reported at baseline by the teacher and included school absence (number of days absent from school in the previous term). Difficulties with reading, spelling or mathematics compared to peers were combined into one variable and categorised as attainment average or above in all three subjects versus some or marked difficulties in one or more of them. Teachers were also asked to estimate “mental age” after the following explanation: “although “mental age” is a crude measure that cannot take account of a child being better in some areas than others, it would be helpful if you could answer the following question: in terms of overall intellectual and scholastic ability, roughly what age level is he or she at?” We divided teacher estimated mental age divided by chronological age, and multiplied the answer by 100 to estimate intellectual ability. From this, we created a binary intellectual ability score based on a cut-off point of 70 [30].

#### Outcome variable

Any psychiatric disorder at follow-up (yes/no) measured using the DAWBA, as described above.

### Statistical analysis

We tested differences in study variables between the analytical sample and those excluded because of incomplete data using independent samples *t *tests for continuous measures and chi-square tests for categorical measures.

The sample was divided into four groups based on the presence of any pLTC or any psychiatric disorder at baseline. As Fig. 1 illustrates, the first two groups explore the *onset* of psychiatric disorder at follow-up among those with and without a pLTC (labelled No pLTC or psychiatric disorder, and pLTC only). Similarly, the second two groups explore the *persistence* of psychiatric disorder among those with or without a pLTC at baseline, which are labelled aspsychiatric disorder only and both pLTC and psychiatric disorder.

Study variables were summarised by the four study groups as means and standard deviations for continuous measures and numbers and percentages for categorical measures. Univariate analyses were first conducted to test for any significant differences in sample characteristics between the four study groups using between-subjects one-way analysis of variance for continuous measures and chi-square tests for categorical measures.

We explored significant predictors of future psychiatric disorders using multivariable binary logistic regression analyses separately for each study group. Predictors included in main models were age, sex, ethnicity, hospitalisation, peer relationship difficulties, parental employment status, housing tenure, family structure, parental mental distress, and family functioning. A substantial proportion of families declined consent for contact with teachers resulting in teacher missing data hence we analysed school factors separately in secondary models.

We conducted a series of sensitivity analyses to test our classification of pLTCs. Specifically, we re-ran main analyses having firstly removed participants with the non-selected conditions from the study (the “no pLTC” groups). Secondly, we expanded the pLTC definition to select CYP with the excluded conditions and re-ran analyses. In addition, we re-ran the main analysis omitting “hospitalisation” in case a strong link with severity overshadowed other associations and, analyses were re-run with the alternate informant data peer relationship difficulties, and for school-related factors reported by teachers. Finally, we examined whether there were significant differences in the proportion of teacher missing data across the four study groups.

All analyses were conducted using SPSS v27 (IBM, Chicago, IL), and were unweighted. Previous analyses on these and other data have shown that adjustment for weighting, stratification and clustering is necessary for point prevalence but not for associations [31, 32].

## Results

### Sample characteristics

Comparisons between the study sample (*N* = 7804) and those excluded because of incomplete data (*n* = 108) revealed that included children were more likely to be of White ethnicity (*V* = 0.18, *p* < 0.001), have fewer peer relationship difficulties (*d* = 0.42, 95% Confidence Interval CI  0.42 to 1.07), have employed parents (*phi* = − 0.08, *p* < 0.001) who own their house (*phi* = − 0.06, *p* < 0.001) and were less distressed (*d* = 0.47, CI   0.73 to 2.24), and to live in a two-parent family (*V* = 0.04, *p* = 0.002). Additionally, excluded participants were more likely to have a diagnosed psychiatric disorder at baseline (*phi* = − 0.039, *p* = 0.001) and follow-up (*phi* = − 0.057, *p* < 0.001).

At baseline, 3925 (50%) CYP had no pLTC or psychiatric disorder, 2931 (38%) CYP had at least on pLTC and no psychiatric disorder, 442 (6%) CYP had at least one psychiatric disorder and no pLTC, and 506 (6%) CYP at least one pLTC and at least one psychiatric disorder (Table 1). As illustrated by Table 1, there were significant differences between the four study groups in age, sex, hospitalisation, peer relationship difficulties, parental employment status, housing tenure, parental mental distress, and family functioning (all *p* values < 0.001), suggesting that CYP with no health conditions face less adversity. Similarly, CYP with no pLTCs nor psychiatric disorders had more favourable school-related characteristics (all *p* values < 0.001).

**Table 1:** Sample characteristics at baseline by the four study groups

Baseline characteristic	No pLTC or psychiatric disorder (*n* = 3925)	pLTC only (*n* = 2931)	Psychiatric disorder only (*n* = 442)	pLTC and psychiatric disorder (*n* = 506)	Group differences
	*n* (%) or M ± SD	*n* (%) or M ± SD	*n* (%) or M ± SD	*n* (%) or M ± SD	Effect size*, p* value
Age [years]	10.07 (3.29)	10.22 (3.24)	10.56 (3.27)	10.60 (3.11)	*η*^2^_p_ = 0.002*, p* < **0.001**
Sex [female]	2024 (51.6)	1381 (47.1)	178 (40.3)	191 (37.7)	*V* = 0.082,* p* < **0.001**
Ethnicity					
White	3604 (91.8)	2671 (91.1)	415 (93.9)	476 (94.1)	*V* = 0.025, *p* = 0.086
Black	91 (2.3)	89 (3.0)	8 (1.8)	15 (3.0)	
Indian, Pakistani or Bangladeshi	143 (3.6)	101 (3.4)	10 (2.3)	7 (1.4)	
Other ethnic group	87 (2.2)	70 (2.4)	9 (2.0)	8 (1.6)	
Hospital stay because of serious illness [yes]	443 (11.3)	568 (19.4)	80 (18.1)	157 (31.0)	*V* = 0.150, *p* < **0.001**
Peer relationships [score]	1.06 (1.31)	1.30 (1.50)	2.91 (2.26)	3.28 (2.38)	*η*^2^_p_ = 0.151, *p* < **0.001**
Parental employment status [active]	3001 (76.5)	2200 (77.3)	275 (62.2)	319 (63.0)	*V* = 0.100, *p* < **0.001**
Housing tenure [own]	3109 (79.2)	2274 (77.3)	243 (55.0)	272 (53.8)	*V* = 0.184, *p* < **0.001**
Family structure					
Traditional family	2813 (71.7)	2094 (71.4)	223 (50.5)	259 (51.2)	*V* = 0.106, *p* < **0.001**
Blended family	400 (10.2)	296 (10.1)	78 (17.6)	69 (13.6)	
Lone parent family	712 (18.1)	541 (18.5)	141 (31.9)	178 (35.2)	
Parental mental health [score]	1.40 (2.43)	1.54 (2.50)	3.17 (3.50)	3.37 (3.57)	*η*^2^_p_ = 0.050, *p* < **0.001**
Family functioning [healthy]	3327 (84.8)	2503 (85.4)	311 (70.4)	341 (67.4)	*V* = 0.142, *p* < **0.001**
School absence^a^					
No days absent	737 (34.4)	489 (31.4)	59 (24.7)	66 (24.3)	*V* = 0.117, *p* < **0.001**
1–5 days	1011 (47.2)	755 (48.5)	91 (38.1)	100 (36.8)	
6–10 days	261 (12.2)	218 (14.0)	40 (16.7)	55 (20.2)	
11–15 days	75 (3.5)	51 (3.3)	13 (5.4)	19 (7.0)	
16 or more days	59 (2.8)	45 (2.9)	36 (15.1)	32 (11.8)	
Educational attainment^a^ [average or above]	1562 (72.9)	1118 (71.8)	100 (41.8)	110 (36.8)	*V* = 0.235, *p* < **0.001**
Intellectual ability^a^ [score ≥ 70]	2112 (98.6)	1527 (98.0)	219 (91.6)	239 (87.9)	*V* = 0.179, *p* < **0.001**

Univariate associations were tested with one-way analysis of variance and chi-square tests

Bold text indicates a statistically significant result

*p*LTC = long-term physical health condition

^a^No pLTC or psychiatric disorder: *n* = 2143; pLTC only: *n* = 1558; psychiatric disorder only: *n* = 239; pLTC and psychiatric disorder: *n* = 272

### Risk factors for the onset of any mental health condition in CYP with and without pLTCs

In CYP with no pLTCs nor psychiatric disorders at baseline, only elevated peer relationship difficulties predicted the onset of any psychiatric condition at the 3-year follow-up (adjusted Odds Ratio [aOR] = 1.29, 95% CI  1.16 to 1.42; Table 2). In CYP with pLTC and no psychiatric disorder at baseline, greater peer relationship difficulties (aOR = 1.29, 95% CI  1.19 to 1.39) also predicted the development of future disorder, along with rented housing (aOR = 1.42, 95% CI  1.01 to 1.99), non-traditional family structure (blended vs. traditional family: aOR = 2.08, 95% CI  1.42 to 3.05), and increased parental mental distress (aOR = 1.09, 95% CI  1.04 to 1.14) (Table 2).

**Table 2:** Multivariable logistic regressions predicting any psychiatric disorder at 3-year follow-up in the four study groups

Model	Associations with the onset of any psychiatric disorder at follow-up adjusted OR (95% CI)		Associations with the persistence of any psychiatric disorder at follow-up adjusted OR (95% CI)	
	No pLTC or psychiatric disorder (*n* = 3925)	pLTC only (*n* = 2931)	Psychiatric disorder only (*n* = 442)	pLTC and psychiatric disorder (*n* = 506)
Age [years]	1.01 (0.96 to 1.05)	1.03 (0.99 to 1.08)	0.96 (0.90 to 1.03)	0.98 (0.92 to 1.04)
Sex [ref. cat. male]	0.95 (0.69 to 1.30)	1.05 (0.79 to 1.39)	**0.56 (0.37 to 0.87)**	0.68 (0.46 to 1.00)
Ethnicity [ref. cat. White]				
Black	0.72 (0.22 to 2.32)	0.88 (0.39 to 1.98)	0.26 (0.04 to 1.58)	2.44 (0.75 to 7.94)
Indian, Pakistani or Bangladeshi	0.59 (0.21 to 1.65)	0.31 (0.10 to 1.02)	1.12 (0.29 to 4.28)	0.74 (0.15 to 3.56)
Other ethnic group	1.65 (0.70 to 3.89)	1.66 (0.24 to 1.86)	1.84 (0.40 to 8.59)	0.85 (0.20 to 3.73)
Hospital stay because of serious illness [ref. cat. yes]	0.89 (0.56 to 1.42)	0.94 (0.67 to 1.32)	1.30 (0.75 to 2.22)	1.08 (0.72 to 1.62)
Peer relationships [score]	**1.29 (1.16 to 1.42)**	**1.29 (1.19 to 1.39)**	**1.19 (1.08 to 1.31)**	**1.27 (1.17 to 1.38)**
Parental employment status [ref. cat. active]	1.27 (0.89 to 1.83)	1.24 (0.98 to 1.84)	1.18 (0.75 to 1.84)	1.23 (0.81 to 1.88)
Housing tenure [ref. cat. own]	1.32 (0.88 to 1.96)	**1.42 (1.01 to 1.99)**	1.22 (0.76 to 1.95)	1.02 (0.66 to 1.58)
Family structure [ref. cat. traditional family]				
Blended family	1.46 (0.91 to 2.33)	**2.08 (1.42 to 3.05)**	1.54 (0.86 to 2.77)	1.13 (0.64 to 1.99)
Lone parent family	1.14 (0.74 to 1.76)	1.18 (0.81 to 1.71)	**1.81 (1.08 to 3.04)**	0.66 (0.41 to 1.04)
Parental mental health [score]	0.99 (0.93 to 1.06)	**1.09 (1.04 to 1.14)**	**1.07 (1.01 to 1.14)**	1.00 (0.94 to 1.05)
Family functioning [ref. cat. healthy]	1.06 (0.67 to 1.63)	1.15 (0.80 to 1.66)	**2.09 (1.32 to 3.31)**	0.94 (0.62 to 1.42)
***R***^**2**^** Tjur**	0.01	0.05	0.15	0.10
**AIC**	1363.77	1525.49	559.39	677.95

Bold text indicates a statistically significant result

*CI* confidence interval, *LTC* long-term condition, *OR* odds ratio, *ref. cat*. reference category

### Risk factors for the persistence of mental health conditions in CYP with and without pLTCs

In CYP with at least one psychiatric disorder but no pLTCs at baseline, increased peer relationship difficulties (aOR = 1.19, 95% CI  1.08 to 1.31), non-traditional family structure (lone parent vs. traditional family: aOR = 1.81, 95% CI  1.08 to 3.03), increased parental mental distress (aOR = 1.07, 95% CI  1.01 to 1.14), and unhealthy family functioning (aOR = 2.09, 95% CI  1.32 to 3.31) predicted the persistence of the disorder follow-up, whereas girls were less likely than boys to experience persistent disorder (aOR = 0.56, 95% CI  0.37 to 0.87; Table 2). In CYP with comorbid physical and mental health conditions at baseline, increased peer relationship difficulties showed a significant effect on the risk of persistent psychiatric disorder three years later (aOR = 1.27, 95% CI  1.71 to 1.38). No other significant findings were detected (Table 2).

### Sensitivity analyses (online resource 2)

Removing all participants with the excluded conditions from the two groups classified without a pLTC did not alter our main findings. When the additional conditions were included in the groups with baseline pLTCs, ethnicity was a significant predictor in pLTC only group. Re-analysis omitting the variable “hospitalization” also obtained the same results. When we replaced information retrieved by alternate responders, teacher reports and child reports of peer relationship difficulties were no longer significant predictors of persistent psychiatric disorder in psychiatric disorder only group, and similarly teacher-reported peer relationship difficulties did not predict the persistence of the disorder in both pLTC and psychiatric disorder group. We also tested the proportion of teacher missing data across the groups and found no significant differences.

In our secondary model with school-related factors included as additional covariates, we found that ethnicity (other vs. White: aOR = 3.78, 95% CI  1.41 to 10.15), renting the house (aOR = 1.92, 95% CI  1.09 to 3.37), and lower intellectual ability (aOR = 8.07, 95% CI  3.13 to 20.83) were additional predictors among children with no pLTC or psychiatric disorder group (*n* = 2122). In pLTC only group (*n* = 1550), the effect of housing tenure (aOR = 1.34, 95% CI  0.83 to 2.18) and parental mental distress (aOR = 1.07, 95% CI  1.00 to 1.15) was diminished, and educational attainment (aOR = 1.67, 95% CI  1.09 to 2.55) was a significant predictor. In psychiatric disorder only group (*n* = 236), the association between parental mental distress and risk of persistent psychiatric disorder was no longer significant (aOR = 1.04, 95% CI  0.94 to 1.14). Results did not change for both pLTC & psychiatric disorder group (*n* = 263).

## Discussion

We explored potential risk factors for the onset and persistence of mental health conditions in CYP with pLTCs compared to those without pLTCs using longitudinal, population-based data. We showed that child, family, and school factors independently predicted psychiatric disorders among CYP with pLTCs, which highlights the importance that these factors are systematically monitored by health care teams.

Parent-reported peer relationship difficulties predicted the risk of psychiatric disorders at follow up in all four groups and was the sole predictor among CYP with comorbid mental and physical health conditions at baseline. Poor peer relationships have been previously linked with persistent Attention Deficit and Hyperactivity Disorder and anxiety disorders at the 3-year follow-up in the same population sample [11] but the latter study did not differentiate CYP with and without pLTCs. Peer relationships may be particularly impacted by the presence of a health condition, either physical or mental, as shown in Table 1, which may limit social activities or involve differences in appearance or behaviour that may ultimately induce negative attitudes by some peers and feelings of exclusion among affected individuals [33]. Self-reported peer relationship difficulties predicted the persistence of psychiatric disorders among children with both pLTC and psychiatric disorder but the failure to detect a relationship in the other groups may relate to lack of power given the smaller number of reports from teachers and young people. Alternatively, parental awareness of peer relationship problems may be an indicator of severity. Similarly, parental mental health was also a significant predictor in groups of children with a physical or a mental health condition at baseline.

Our findings suggest that primary care, paediatric, and mental health specialist teams should systematically ask about friendships and parental mental health. For example, the Strengths and Difficulties Questionnaire has been suggested for inclusion in both CAMHS [34] and paediatric primary care screening [35] to delineate broad categories of behavioural, and emotional concerns, including peer problems. Equally, educators and third sector organisations working with CYP should pay close attention to peer interactions and work to develop supportive and positive peer relationships for all children. Bullying casts a shadow over current and future mental health and is arguably the most tractable public mental health risk factor [36].

As suspected, we observed some distinct characteristics predicting the onset of poor mental health in CYP with pLTCs compared to their physically healthier peers. Notably, more factors predicted the onset of disorder among children with pLTCs, which included rented housing, blended family structure, and parental mental distress, in addition to peer relationship difficulties. This suggests that paediatricians should actively monitor the mental health of their patients and their parents, and should encourage mental health treatment for either or both, if required. Indeed, given the bi-directional effect between parental and child mental health, successful intervention with one might protect the mental health of other family members [1, 16, 37–39]. A scoping review summarized the findings of 18 studies and reported that the prevalence of parental mental illness among children served by CAMHS widely ranged from 16 to 79%. The study also identified several limitations of assessing parental mental health in CAMHS [40]. For example, some case-note audits did not include information on whether the parental mental issues were diagnosed; some parents might be reluctant to disclose their own mental health issues with CAMHS clinicians while their child was present, which may affect the completeness and accuracy of the measurement. To better serve the family as a whole, researchers have suggested an inter-agency collaboration between child and adult mental health services [41].

Only poor peer relationships predicted persistent mental health conditions among CYP with pLTCs, compared with several other factors for their physically healthier peers. Various family factors have been associated with the risk of persistent psychopathology in CYP without pLTCs, suggesting that family factors are particularly impactful in CYP struggling with their mental health. This emphasizes the need to support families where children have additional health needs, either physical or mental. Socioeconomic factors, family structure, and parental mental health have been previously associated with poor mental health among CYP in population-based analyses [12, 16, 17]. The differences in predictors of onset and persistence of psychiatric disorders between CYP with pLTCs and the rest of the school-age population suggest variations in the aetiology of psychopathology, which future studies may wish to enrich the sample to investigate school-related or other factors, which will require samples that provide adequate power.

In secondary models, intellectual ability and educational attainment independently predicted the risk of new onset psychiatric disorders in the no pLTC or psychiatric disorder and pLTC only group, respectively, suggesting that schools need to ensure adequate support for pupils who struggle with learning. School-related factors have largely been ignored in studies of the mental health of CYP with poor physical health, so our findings need to be replicated in other samples.

High comorbidity rates between chronic physical health conditions and psychopathology have been well established [1–13]. Our study adds to the evidence base by suggesting that multiple family and school factors independently predicted psychiatric disorders among CYP with pLTCs. Our findings support previous recommendation for an integrated care approach that go beyond the physical and mental health care to involve other important systems [42]. We call for a much wider collaboration to meet the varied needs of CYP with pLTCs. Other significant roles, such as peers, teachers, and parents, should also be involved in the integrated care framework.

This study benefitted from a large, carefully selected population-based sample, and robust measures of mental health and longitudinal data, which allowed us to elucidate the temporal relationship between baseline factors and future psychopathology. However, we are assuming “persistence” in psychopathology from two surveys spaced three years apart; ideally, we would have more data points and shorter gaps between them. Persistence could, in some cases (e.g. depression), reflect relapse after period(s) of recovery but we did not test this as we lacked sufficient numbers of cases to study psychiatric disorders separately and interim data. As expected in population-based studies, a relatively small number of CYP fulfilled diagnostic criteria for psychiatric disorder, which prevented us from studying individual pLTCs or disorders separately. As with all secondary analyses, we were constrained by the limits of the initial surveys. For example, other than hospitalization, which is rare, and so may identify particularly severe cases, we lacked data on the severity of pLTCs, which can vary greatly. The inclusion of hospitalization attempted assess the severity of health problems, which may be related to the risk to mental health, as may the duration and severity of parental mental health and family dysfunction, for which we had brief single measures. This was why we excluded physical conditions that are mostly mild and self-limiting. Similarly, teacher estimated mental age is dependent on teacher’s understanding of the concept and their frame of reference in terms of the population of children that they have worked with. Obviously systematic testing would have been preferable for such an important construct. While population-based studies support the study of large and representative samples, their size precludes the highly detailed assessments seen in clinical samples, such as standardized measures of Intellectual Quotient or Development.

We excluded participants with missing data from this study, but those with missing data were more likely to be in poor mental health and to have more risk factors (16). The exclusion of those potentially at most risk may have diminished the likelihood of detecting an association but suggests that positive associations we reported are likely to be robust. Age, sex, ethnicity, hospitalisations, and family functioning were not associated with the onset of psychopathology among CYP with pLTCs in our analyses, although all have been significantly linked with mental health outcomes in previous studies [4–9]. We may have lacked statistical power to detect some effects; for example, hospitalisation, which was relatively rare. Also, our population-based sample reflected the ethnic composition of England with few children from some minority groups. Alternatively, such effects may operate at the level of individual pLTCs or individual psychiatric disorders that our broad analysis may have masked, as suggested by previous studies examining specified conditions, for instance depression in cancer patients. The assessment of pLTCs and study predictors was based on parental-, teacher- or self-report which may involve information or recall bias; future research should corroborate these with administrative health and education data. Indeed, the analysis of secondary data is always limited to the available variables, and we lacked information on the severity and duration of pLTCs which may be important and should be explored in future studies.

In conclusion, many of the risk factors elicited, such as peer relationships and parental mental ill-health, are potentially tractable and may be useful indicators of patients who require mental health interventions, if the findings are replicated in clinical samples.
